# Supplementary material for: Is End-Stage Renal Disease Tumor Suppressive? Dispelling the Myths
Source: Cancers (Basel). 2024 Sep 12;16(18):3135. doi: 10.3390/cancers16183135 (PMC11430482; doi:10.3390/cancers16183135)
Supplement: Supplementary file 1 [file cancers-16-03135-s001.zip › cancers-3173322-supplementary.pdf]

Table S1. Common cancers and their risk among patients with ESRD undergoing hemodialysis

Rank	Cancer site (proportion, %)	SIR or pooled SIR (95% CI)	References
1	Lung (11.6%)	0.98 (0.77–1.24)	2, 8, 26
		0.94 (0.63–1.34)	22
		1.5 (1.4–1.7)	23
		0.916 (0.395–1.805)	24
		1.09 (0.74–1.55)	25
2	Breast (11.6%)	1.15 (0.9–1.46)	2, 8, 26
		1.65 (0.97–2.60)	22
		1.3 (1.0–1.5)	23
		0.81 (0.42–1.41)	24
3	Colorectum (10.2%)	1.13 (0.90–1.43)	2, 8, 26
		1.53 (1.11–2.05)	22
		1.3 (1.1–1.5)	23
		0.607 (0.365–0.948)*	24
		0.85 (0.56-1.24)	25
4	Prostate (7.1%)	0.87 (0.69–1.09)	2, 8, 26
		0.27 (0.05–0.79)	22
		0.9 (0.8–1.0)	23
		1.023 (0.375–2.227)**	24
		0.89 (0.62–1.23)	25
5	Stomach (5.7%)	1.03 (0.71–1.50)	2, 8, 26
		1.10 (0.47–2.17)	22
		1.5 (1.1–2.2)	23
		0.607 (0.365–0.948)*	24
		1.20 (0.66–2.02)	25
6	Liver (4.7%)	1.39 (1.28–1.51)	2, 8, 26
		1.25 (0.68–2.09)	22

		2.0 (1.4–3.0)	23
		0.607 (0.365–0.948)*	24
		1.53 (0.83–2.56)	25
7	Esophagus (3.2%)	1.9 (1.3-2.7)	23
8	Cervix uteri (3.2%)	1.76 (1.09–2.86)	2, 8, 26
		4.12 (1.65–8.48)	22
		2.0 (1.2–3.5)	23
		1.023 (0.375–2.227)**	24
		7.0 (0.85–25.30)	25
9	Thyroid (3.1%)	4.92 (1.43–16.93)	9, 26
		3.43 (1.25–7.46)	22
		3.0 (1.7–5.5)	23
		0.809 (0.349–1.594)***	24
		1.39 (0.17–5.01)	25
10	Bladder (3.0%)	2.51 (1.85–3.41)	2, 8, 26
		2.50 (1.14–4.75)	22
		1.8 (1.5–2.1)	23
		4.691 (2.424–8.195)****	24
		0.94 (0.54–1.52)	25

Data not shown for ranks 11 to 13

14	Kidney (2.2%)	4.87 (4.14–5.72)	2, 8, 26
		12.28 (8.44–17.08)	22
		2.8 (2.2–3.7)	23
		4.691 (2.424–8.195)****	24
		3.18 (2.06–4.69)	25

* Digestive organs, including the stomach, colon/rectum, liver, and pancreatobiliary and other gastrointestinal tracts.

**Reproductive organs, including the uterus and prostate gland.

***Endocrine organs, including the thyroid.

****Urinary tract, including the kidneys and bladder.

Abbreviations: CI, confidence interval; ESRD, end-stage renal disease; SIR, standardized incidence ratio
